# Novel inhibitors of *Mycobacterium tuberculosis* GuaB2 identified by a target based high-throughput phenotypic screen

**DOI:** 10.1038/srep38986

**Published:** 2016-12-16

**Authors:** Jonathan A. G. Cox, Grace Mugumbate, Laura Vela-Glez Del Peral, Monika Jankute, Katherine A. Abrahams, Peter Jervis, Stefan Jackenkroll, Arancha Perez, Carlos Alemparte, Jorge Esquivias, Joël Lelièvre, Fernando Ramon, David Barros, Lluis Ballell, Gurdyal S. Besra

**Affiliations:** 1 Life and Health Sciences, Aston University, Aston Triangle, Birmingham B4 7ET, UK; 2 European Molecular Biology Laboratory, European Bioinformatics Institute (EMBL-EBI), Wellcome Trust Genome Campus, Hinxton, Cambridge, United Kingdom, CB10 1SD; 3 Molecular Discovery Research, GlaxoSmithKline, Santiago Grisolia 4, 28760 Tres Cantos, Madrid, Spain; 4 School of Biosciences, University of Birmingham, Edgbaston, Birmingham B15 2TT, UK; 5 Diseases of the Developing World, GlaxoSmithKline, Severo Ochoa 2, 28760 Tres Cantos, Madrid, Spain

## Abstract

High-throughput phenotypic screens have re-emerged as screening tools in antibiotic discovery. The advent of such technologies has rapidly accelerated the identification of ‘hit’ compounds. A pre-requisite to medicinal chemistry optimisation programmes required to improve the drug-like properties of a ‘hit’ molecule is identification of its mode of action. Herein, we have combined phenotypic screening with a biased target-specific screen. The inosine monophosphate dehydrogenase (IMPDH) protein GuaB2 has been identified as a drugable target in *Mycobacterium tuberculosis*, however previously identified compounds lack the desired characteristics necessary for further development into lead-like molecules. This study has identified 7 new chemical series from a high-throughput resistance-based phenotypic screen using *Mycobacterium bovis* BCG over-expressing GuaB2. Hit compounds were identified in a single shot high-throughput screen, validated by dose response and subjected to further biochemical analysis. The compounds were also assessed using molecular docking experiments, providing a platform for their further optimisation using medicinal chemistry. This work demonstrates the versatility and potential of GuaB2 as an anti-tubercular drug target.

Tuberculosis (TB), the disease caused by the pathogen *Mycobacterium tuberculosis (Mtb*), affects 9 million people each year, and is attributed to over 1.5 million deaths, and is reported to be the biggest killer worldwide due to a single infectious agent alongside HIV/AIDS^1^. This, coupled with the emergence of multi-drug and extensively-drug resistant (MDR and XDR) *Mtb* infections, which infects approximately 0.5 million people each year, has prompted efforts to produce new and safe antibiotics that do not interfere with current anti-retroviral drugs in HIV/TB co-infected individuals^2^. The current WHO-recommended directly observed treatment short-course (DOTS) regimen for drug-susceptible infections of *Mtb* requires further refinement due to the lengthy duration of treatment and considerable pill burden^5^.

Recent innovations in high-throughput screening (HTS) have accelerated drug discovery, making it possible to test millions of compounds for antimicrobial activity in weeks rather than years. As a result many new molecules have been discovered by whole-cell phenotypic screening campaigns and published due to their potent inhibitory activity. Indeed, the first new TB drug, Bedaquiline (brand name Sirturo^TM^, formerly TMC207) to receive FDA approval since 1971, was first recognized as a potential anti-TB compound in a whole-cell phenotypic HTS campaign. Sirturo^TM^, a member of the diarylquinoline (DARQ) family, was identified as an inhibitor of both drug-sensitive and drug-resistant *Mtb*^6^,^7^. During clinical trials, it was found that many patients experienced drug-induced QTcF prolongation (an extended time between depolarization and repolarization of the ventricles) as a side-effect of Sirturo^TM^. Nevertheless, the drug was approved for use on a case-by-case basis in the treatment of MDR-TB^8^. Despite the novelty of phenotypic ‘hits’, the majority of compounds are unlikely to progress as leads or drug candidates due to their undesirable molecular structure, promiscuous mode of action and their frequent appearance in screening campaigns. Some of these molecules are now considered artefacts of HTS, and have recently received the designation as PAINS (Pan-Assay InterferenNe compoundS), and filters are being employed to prevent time and money being invested on molecules of this type^9^.

Inosine monophosphate (IMP) is a metabolic precursor in the biosynthesis of the purine nucleotides adenine and guanine. Guanosine monophosphate (GMP) requires the synthesis of xanthosine monophosphate (XMP) from IMP by the inosine monophosphate dehydrogenase (IMPDH), of which there are three in *Mtb,* GuaB1 (Rv1843c), GuaB2 (Rv3411c) and GuaB3 (Rv3410c)^10^,^11^. The rate limiting two-step catalysis of IMP to XMP conversion by IMPDH requires nicotinamide adenine dinucleotide (NAD^+^) as a cofactor, which is first reduced to NADH by a dehydrogenation reaction, forming an intermediate covalent bond between IMPDH and XMP, followed by a hydrolysis reaction that breaks the covalent attachment and releases XMP^12^. GuaB2 is the only catalytically active and essential IMPDH shown by transposon site hybridization (TraSH) to be required for viability of *Mtb*^13^,^14^. Inhibition of GuaB2 results in depletion in guanine nucleotides for DNA and RNA synthesis^15^,^16^. Biochemical characterisation of GuaB2 has been previously conducted and a series of diphenyl-urea (DPU) based inhibitors of *Mtb* were shown to inhibit *Mtb* GuaB2. An over-expresser based MIC shift analysis was conducted to validate the target in *Mycobacterium smegmatis* and a 16-fold increase in MIC (0.5–8 μg.ml^−1^) was observed^13^. There are three distinct classes of IMPDH inhibitors, type I inhibitors are IMP or XMP analogues, type II inhibitors are NAD^+^/NADH analogues, whereas type III are muti-substrate inhibitors^17^. The aforementioned DPU compounds were designated as type III, along with a series of compounds identified in a screen of commercially available molecules which target mycobacterial GuaB2. In this instance, the lead molecule, 7759844, demonstrated high potency with a K_i_ 0.603 μM and MIC of 0.633 μg.ml^−1^, however displayed toxicity in a chronic mouse model^18^. Several crystal structures of GuaB2 from *Mtb* have recently been determined in complex with substrate, product and cofactors along with a number of new compounds with anti-mycobacterial activity^19^,^20^,^21^. This enhanced understanding of the biophysics of GuaB2 inhibition can be used for *in silico* drug discovery and for the assessment of newly discovered anti-mycobacterial compounds targeting GuaB2. Following a high-throughput resistance-based phenotypic screen using a GuaB2 over-expressing strain of *M. bovis* BCG, we present 7 new inhibitors of *Mtb* GuaB2, and their subsequent biochemical and *in silico* assessment. These diverse compounds contribute to a growing pool of GuaB2 inhibitors, laying a foundation for a future medicinal chemistry optimisation programmes and acceleration of GuaB2 as a future anti-tubercular drug target.

## Results

### Identification and *in vitro* validation of novel inhibitors of *Mtb* GuaB2

**Figure 1:**
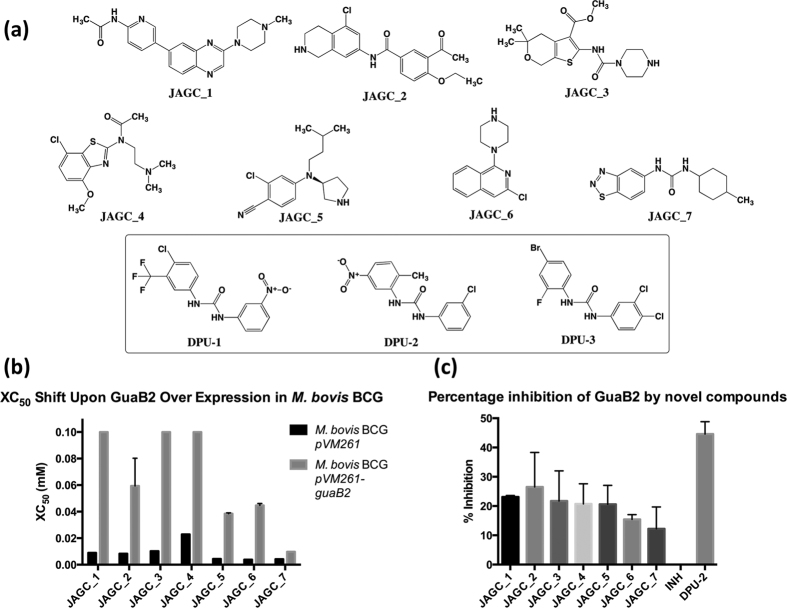
(**a**) Newly identified anti-mycobacterial compounds that target the essential inosine monophosphate dehydrogenase, GuaB2 (JAGC_1–7). These molecules have been identified in a target-based HTS using *M. bovis* BCG over-expressing GuaB2 from *Mtb.* Chemical structures of the DPU compounds (DPU-1-3) are also shown (boxed)^13^. Details of the chemical data for JAGC_1–7 is provided in Supplementary Table 1. (**b**) Graph illustrating the difference in XC_50_ (compound concentration required to inhibit cell viability by 50%) of each compound against *M. bovis* BCG pMV261 and *M. bovis* BCG pMV261-*guaB2*. (**c**) Graph showing the percentage biochemical inhibition of GuaB2 by JAGC_1–7 respectively, with isoniazid (INH) and an optimized diphenyl urea compound (DPU-2), as respective negative and positive controls.

Here we present 7 novel inhibitory compounds targeting *Mtb* GuaB2. These compounds have been given the identifier JAGC_1 to JAGC_7, respectively. The chemical structures of each of these molecules are given in Fig. 1(a) and details of their chemical data are presented in Supplementary Table 1.

These new GuaB2 anti-mycobacterial compounds were discovered by a target-specific HTS using *M. bovis* BCG over-expressing the essential IMPDH, against a GSK 10,000 compound collection of known inhibitors of *Mtb* (the ‘TB Box’ set)^22^. A commercially available luciferase reporter assay was used to measure cell viability of each strain following 7 days of incubation with each of the ‘TB Box’ set at 1 and 10 μM in a 1536-well format. Hits were identified based on shift in apparent inhibition (calculated as % inhibition of *M. bovis* BCG pMV261 (empty plasmid) *minus* % inhibition of *M. bovis* BCG pMV261-*guaB2* [based upon duplicate data]), resulting in 256 compounds that were further selected to be tested in a dose response manner.

Compounds were analysed using a serial dilution from 10 mM to 0.17 nM against *M. bovis* BCG pMV261 and *M. bovis* BCG pMV261-*guaB2.* The percentage inhibition at each concentration was used to determine the XC_50_ (compound concentration required to inhibit cell viability by 50%) of each compound against the over-expresser and empty vector strains based on duplicate data. This resulted in the identification of 66 compounds with a significant shift in XC_50_ (pXC_50_ ≥ 0.3), representing a 25.78% successful refinement from the initial HTS. The XC_50_ data for *M. bovis* BCG pMV261 and *M. bovis* BCG pMV261-*guaB2* against JAGC_1–7 is shown in Fig. 1(b). The pXC_50_ values for JAGC_1–7 are displayed in Table 1 along with the Z’ values for the dose response validation. The 66 dose response validated molecules were then subjected to a PAINS filter in order to remove compounds that are likely to represent promiscuous hits^9^. From this, 19 compounds were identified that displayed phenotypic resistance upon GuaB2 over-expression in *M. bovis* BCG when compared to an empty vector control strain. However, in order to rule out the potential of compound metabolism by *M. bovis* BCG, resulting in toxic adducts with specificity to *Mtb* GuaB2 (i.e. GuaB2 targeting pro-drugs), the 19 compounds were re-tested in a *Mtb* GuaB2 *in vitro* biochemical assay^18^. The optimised DPU compound DPU-2 and fatty acid biosynthesis inhibitor isoniazid (INH) were used as positive and negative controls of *Mtb* GuaB2 inhibition, respectively. The results of the *in vitro* biochemical assay identified 7 compounds with mean inhibitory activity against GuaB2 ranging from 26.5% (JAGC_2) to 12.3% (JAGC_6) inhibition, compared to the optimised DPU-2 compound that exhibited 44.6% inhibition and INH which appeared to enhance GuaB2 activity (−9% inhibition). The *in vitro* biochemical assay data is summarised in Fig. 1(c) and is detailed in Table 1.

### *In silico* Binding of Compounds JAGC_1–7 and DPU-2 to *Mtb* GuaB2

We performed docking calculations to assess the binding orientations of compounds JAGC_1–7 and to study their protein-ligand interactions using the Internal Coordinate Mechanism (ICM) method developed by Molsoft L.L.C^23^. Molecular docking approaches are widely applied in drug discovery to provide information that facilitates structure-based drug discovery and molecular modifications. The 4ZQP GuaB2-KP3 complex regenerated the crystal structure conformations of most ligands during cross-docking. Therefore, the structure was superior over the rest of the complexes (PDB codes: 4ZQO and 4ZQN, 4ZQM) extracted from the PDBe, http://www.ebi.ac.uk/pdbe^24^. The seven compounds, JAGC_1–7, and a previously documented optimised inhibitor of GuaB2, DPU-2^13^ were docked into the NAD^+^ and IMP binding sites based on two crystal structures (4ZQP and 4ZQM). The structure, 4ZQP, is a complex of *Mtb* GuaB2 with two ligands bound, IMP and an inhibitor KP3 occupying the NAD^+^ site, whereas 4ZQM shows GuaB2 co-crystallised with the product XMP and the co-factor NAD^+^. All compounds demonstrated higher affinities for the NAD^+^ site as shown by the highly negative ICM scores (Table 2). When compared to the affinity of the crystal structure ligand, KP3, two compounds JAGC_2 and JAGC_7 displayed higher binding affinity and all compounds had lower affinity for the IMP site compared to XMP.

**Figure 2:**
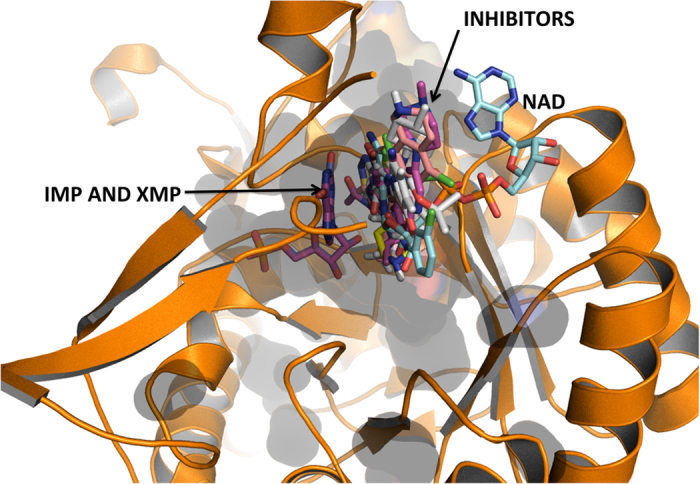
An overlay of the *in silico*-derived binding orientations of all JAGC compounds, indicating that the compounds favour the NAD binding site of GuaB2. Their aromatic rings are positioned in the NAD^+^ nicotinamide sub-site close to the IMP and XMP binding sites.

The NAD^+^ binding site in *Mtb* GuaB2 stretches from the adenosine sub-site defined by N289, L291, V292 and G260 to the nicotinamide sub-site with the middle part of the molecule stretching over the inhibitor minimal structural moiety (IMSM)^19^. Occupation of the nicotinamide sub-site and interactions of small molecules with the purine ring of IMP have been found to be crucial for binding and protein inhibition. All seven compounds consist of at least one aromatic ring or cyclic sub-structure and their highest binding affinity conformations indicated that one ring occupied the nicotinamide sub-site resulting in π-stacking with the purine ring in IMP (Fig. 2). The molecular orientations in Fig. 2 gave the highest affinities for binding and their positions relative to IMP and NAD^+^ are shown. Compound JAGC_7 showed the highest Ligand Efficiency Index (LEI = 0.99) for the NAD^+^ site and for the IMP site (LEI = 0.64) (Table 2).

### *In silico* Binding Mode of JAGC_7

**Figure 3:**
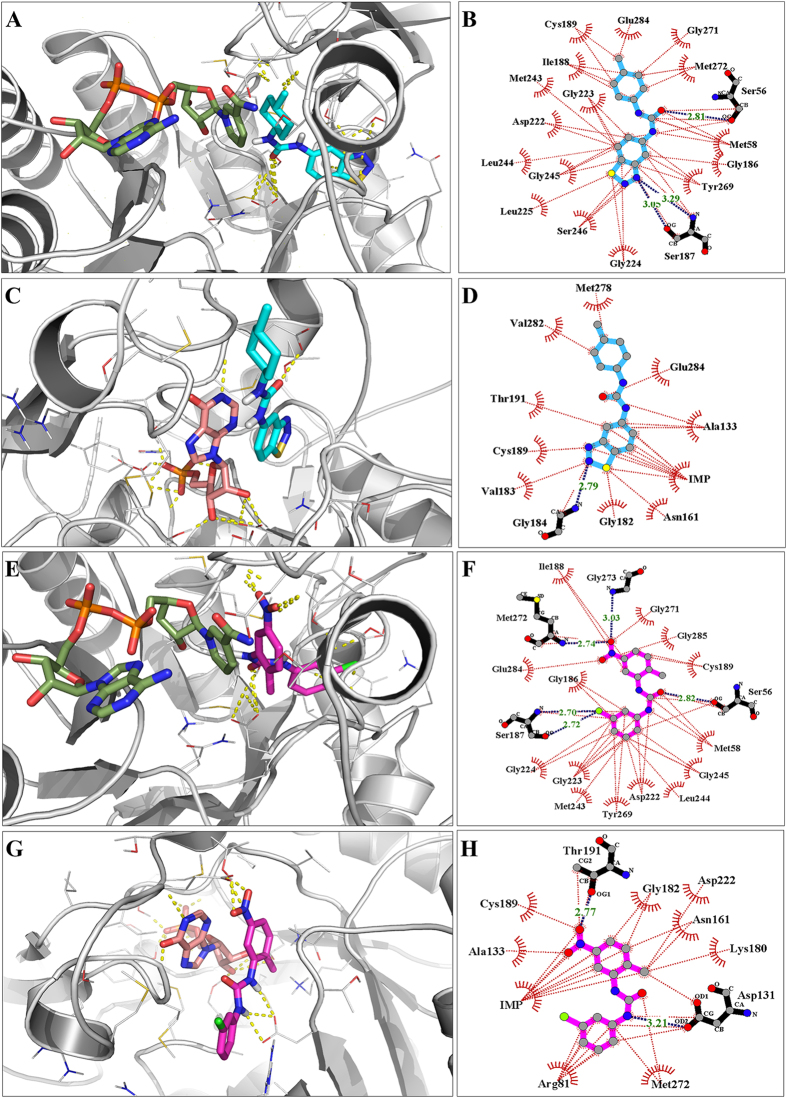
Binding orientations of compounds JAGC_7 and DPU-2 in the binding pocket of GuaB2. (**A** and **B**) Orientation of JAGC_7 (cyan) in the IMP site. Its 4-methyl cyclohexane ring occupies the xanthosine subsite and forms π-π stacking with the nicotinamide moiety of NAD^+^ and GuaB2-JAGC_7 interactions. Green sticks represent the NAD^+^ molecule. (**C** and **D**) Orientation of JAGC_7 in the NAD^+^ binding site where the 1,2,3,benzothiadiazole ring occupies the nicotinamide position and forms π-π bonds with IMP (brown sticks), and its interactions with binding site residues. (**E** and **F**) Binding orientation of DPU-2 (magenta) and its interactions of in the IMP binding site. The nitrophenyl moiety is positioned towards the nicotinamide scaffold of NAD^+^. (**G** and **H**) Binding orientation and interactions of DPU-2 in the NAD^+^ binding site. The nitrophenyl group is π stacking with the purin-6-one ring. In the interaction figures B, D, F and H, the red dotted lines represent hydrophobic interactions including π-π interactions between the inhibitor and the residues in the GuaB2 binding pockets and hydrogen bonds are shown by the dashed blue line.

Compound JAGC_7, (3-(1,2,3,-benzothiadiazol-5-yl)-1-(4-methylcyclohexyl)-urea, has two ring systems and in the IMP site, the methylcyclohexane ring is placed either in the xanthosine or purine sub-site, whilst the benzothiadiazole ring orientates towards the IMP/XMP phosphate group sub-site (Fig. 3A). Similar to IMP and XMP, in this position the molecule is anchored by a dense network of hydrophobic interactions involving aromatic rings, cyclohexane and more than ten residues that include Tyr269, Met272 and Leu244 (Fig. 3B). This orientation places the cyclohexane in a stacking position with the π electrons in the nicotinamide end of NAD^+^ anchored the molecule resulting in even stronger carbon- π interactions. In addition, there are hydrogen bonds involving the nitrogen atoms in the thiadiazole ring with Ser187, and the Urea Oxygen with Ser56, which stabilise the other end of the molecule. The compound showed better binding affinity in the NAD^+^ site where the aromatic benzothiadiazole ring, was placed in the nicotinamide site and was involved in π stacking with the purine ring in IMP (Fig. 3C). Stability of this conformation is also enhanced by hydrogen bonding to G184 and T343 whilst the cyclohexane ring is placed over the IMSM moiety. There are multiple hydrophobic interactions that due to π-π stacking between IMP and the 1,2,3-benzothiazol-5-yl rings (Fig. 3D).

### *In silico* Binding Mode of DPU-2

The binding mode of the optimised DPU compound DPU-2, a known inhibitor of *Mtb* GuaB2^13^, was similar to that of JAGC_7 and the rest of the compounds. When docked into the IMP site the nitrophenyl ring was placed in the xanthosine sub-site and there was π-π stacking with the nicotinamide ring in NAD^+^ (Fig. 3E) and hydrogen bonds to Met272 and Gly273 (Fig. 3F). The chlorophenyl ring occupied the phosphate group sub-site and participated in π interactions with the hydrophobic residues like Gly223, L244, M243, π-π interactions with Tyr269, and hydrogen bonding to S56 through the Urea O atom and S187. However the binding scores in this site (ICM score = −7.21, LEI = 0.34) showed that binding affinity was less than in the NAD^+^ site (ICM score = −16.36, LEI = 0.78). In the NAD^+^ site the methyl nitrophenyl ring π stacked with the purine ring of IMP (Fig. 3G), resulting in multiple hydrophobic interactions, hydrogen bonding with Asp131 and Thr191 in addition to a number of other hydrophobic contacts with the binding site residues (Fig. 3H).

## Discussion

**Figure 4:**
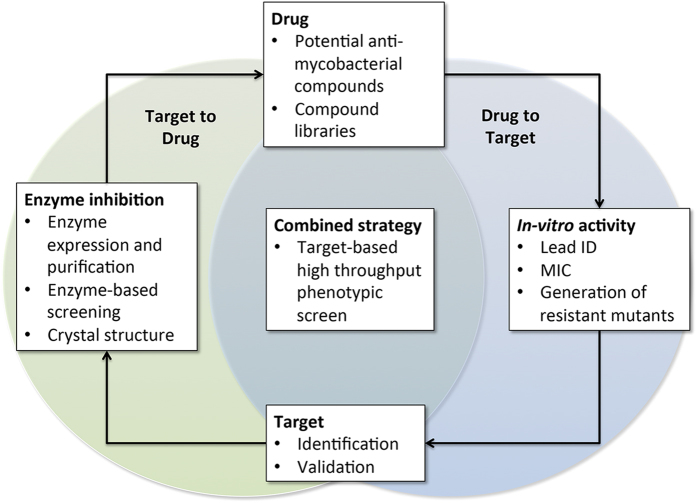
A change in strategy for *Mtb* drug discovery. Recent innovations in screening technologies and robotics have heralded a change in strategy for primary antimicrobial drug discovery. Efforts to design inhibitors of specific bacterial targets as potential drugs (**Target to Drug**) have given way to the screening of substantial compound libraries for whole cell activity and subsequent target identification (**Drug to Target**). The former strategy lacks efficacy, as biochemical inhibitory activity does not necessarily translate to whole cell inhibitory activity. The latter, produces a vast array of antimicrobial compounds, however their mode of action is often not conducive to their progression as clinically relevant drugs. Here we have employed a combined strategy, screening for phenotypic resistance caused by over-expression of a mycobacteria-specific drug target, GuaB2, in *M. bovis* BCG. The outputs of this approach are compounds with confirmed whole cell activity and with a mode of action conducive to further development from compounds to drugs.

Until now, two opposing independent strategies have been favoured for TB drug discovery. The historical target to drug approach necessitates a formative detailed understanding of the selected target. This includes: (i) establishing the *in vivo* essentiality of the target, (ii) biochemical characterisation of the target and (iii) establishing the structures of the targets in complex with substrates and/or cofactors. Only then can inhibitory molecules be screened from compound libraries, designed *de novo*, discovered *in silico*, developed by fragment-based drug design, or rational drug design based upon cofactors or substrate chemical structure. Although compounds may show biochemical inhibition, they are unlikely to show whole-cell activity, and thus a target-to-drug approach is often considered a time consuming and costly drug discovery process. By contrast, a drug-to-target strategy generally involves a phenotypic screening method utilising whole-cells in order to establish potent inhibitors to cell growth and/or cell viability. This strategy has recently become the favoured approach in that it takes advantage of HTS to allow the screening of millions of compounds in a cost effective and timely manner. However, there remain limitations to this approach due to the biasing of compound libraries or the high propensity to identify compounds which target enzymes with highly susceptible binding sites, which may have homology to mammalian enzymes and thus will limit the progression of a hit compound. As a result, there exists a demand for a drug discovery strategy, which combines the specificity of the target to drug approach with the broad reaching chemical potential of the drug to target approach (Fig. 4).

Here we have identified a series of novel compounds with activity against *M. tuberculosis* H37Rv. These hit compounds from the ‘TB Box’ set have been discovered by a phenotypic HTS campaign utilising constitutive over-expression of a previously identified TB drug target, GuaB2, which was previously validated^18^. Upon over-expression of GuaB2, a resistance phenotype was observed that was not apparent in the empty vector control strain. Initial hit compounds have been verified in dose response and hits prioritised according to their pXC_50_ shift data. Additionally, hits have also been validated as inhibitors in an *in vitro Mtb* GuaB2 biochemical assay.

Molecular docking approaches are widely applied in drug discovery and basic science to provide target-ligand complexes and hence information that facilitates structure-based drug discovery and molecular modification. The NAD^+^ binding site in GuaB2 stretches from the adenosine sub-site defined by N289, L291, V292 and G260 to the nicotinamide sub-site with the middle part of the molecule stretching over the inhibitor minimal structural moiety (IMSM)^19^. Occupation of the nicotinamide sub-site and interactions of small molecules with the purine ring of IMP have been found to be crucial for binding and protein inhibition. In this site, the seven hit compounds JAGC_1–7 had had similar binding orientations to that of DPU-2. The observed activities of these compounds are attributed to a favourable positioning of molecular scaffolds in the binding site resulting in the important ligand-IMP interactions. For the hit compounds either an aromatic in JAGC_1–6 and DPU-2 or the hydrophobic cyclohexane ring in JAGC_7 occupied the nicotinamide sub-site, a position that favours π-π stacking with the purine ring in IMP. A closer look at the GuaB2-JAGC_7 interactions reveals that the hydrophobic ring provides numerous interaction points to IMP (Fig. 3D), similarly to the nitrophenyl ring in DPU-2 and IMP interactions (Fig. 3H). The network of hydrophobic bonds coupled with hydrogen bonds stabilise the binding conformations of the ligands and result in high binding affinities. On the other hand, all hit compounds including DPU-2 displayed low affinities for buried IMP/XMP site irrespective of more hydrophobic and polar contacts (Fig. 3B and F) and similar binding orientations.

This new series of inhibitors, JAGC_1–7, together with the knowledge about their target and binding modes, provides a solid platform for future medicinal chemistry efforts to improve the activities of these scaffolds. Furthermore the identification of such a diverse collection of compounds gives credence to the incorporation of target-to-drug based strategies in high-throughput *Mtb* drug discovery.

## Materials and Methods

### Strains, transformation and culture

All experiments were performed in *M. bovis* BCG which has been transformed by a standard heat shock protocol with pMV261, a kanamycin-resistant mycobacterial over-expresser plasmid, which had been modified by addition of *Mtb guaB2*. An empty vector strain (pMV261 only) was also prepared. Transformants were selected on Middlebrook 7H11 mycobacterial media (Difco) supplemented with 10% (v/v) OADC (oleic acid (1.25 × 10^−2^% v/v), albumin (1.25% w/v), dextrose (0.5% w/v) and catalase (1 × 10^−3^% w/v) (Sigma-Aldrich)), containing kanamycin at 25 μg.ml^−1^. Successful transformants were transferred into Middlebrook 7H9 liquid media (Difco) containing kanamycin at 25 μg.ml^−1^, 0.05% (v/v) Tween-80, 0.25% (v/v) glycerol and supplemented with 10% (v/v) ADC (albumin (1.25% w/v), dextrose (0.5% w/v) and catalase (7.5 × 10^−4^% w/v) (Sigma-Aldrich)). Growth rate was monitored by measuring optical density (OD) at 600 nm and cells were passaged to maintain a mid-log OD_600_ of 0.4–0.8. To prevent mutagenesis, no more than 4 passages were conducted before cultures were reinitiated from a glycerol stock.

### High-throughput screening of over-expresser strains in single shot

The initial HTS was conducted in single shot, with 1536 well plates (32 rows × 48 columns) prepared with compounds at 1 and 0.1 mM (50 nl total compound volume). A liquid dispensing robot (Multidrop^TM^ Combi (Thermo Scientific)) was used to dispense 5 μl of a diluted mid-log culture (diluted to OD_600_ 0.125 corresponding to 1 × 10^7^ cfu.ml^−1^) into each well, so compounds were tested at 10 μM and 1 μM final assay concentration. A negative (DMSO only) control (control 1, column 11 and 12) and INH treated positive control (control 2, column 35 and 36) were also prepared on each plate. Plates were hermetically wrapped in aluminium foil with dummy plates at the top and bottom of each stack and incubated for 7 days at 37 °C in 5% CO_2_ with humidity. Following incubation, 5 μl of reconstituted BacTiter-Glo™ was added to each well and the luminescence was measured using a Viewlux Reader (Perkin Elmer). The BacTiter-Glo™ assay causes cell lysis and allows the fluorimetric quantification of the amount of adenosine triphosphate (ATP) present in the reaction mix by an ATP-dependent thermostable luciferase luminescent signal, which is directly proportional to the number of viable cells in the culture. This method was favoured over conventional Resazurin treatment as there is a lesser propensity for false positives. The effect of a given inhibitor was calculated as:


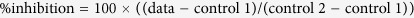


(where control 1 = maximum activity (DMSO only; uninhibited growth), and control 2 = bacterial growth completely inhibited (by treatment with 10 μM isoniazid).

Assay performance statistics (signal to background ratio and Z′) were calculated using templates in ActivityBase XE (ID Bussines Solutions Ltd, Surrey, UK).

(The 256 compounds with the higher shift (calculated as % Inhibition in Empty vector strain - % Inhibition in over-expresser strain) from each strain tested were selected to be tested in dose response.)

### Dose response validation of hits

Hits identified in the single shot HTS were investigated further by conducting a dose response experiment following the same 1536 well format and experimental conditions as described above. The only difference in method is that hit compounds were plated in serial dilution of 11 steps from 10 mM to 0.17 nM using a 1/3 dilution factor all in a 50 nl total volume. The controls as described above were repeated for each plate and data was obtained in duplicate.

### Data analysis

The % Inhibition for each compound concentration tested were calculated as described above. Dose response curves for each of the compounds were analyzed using the ActivityBase XE template (ID Business Solutions Ltd, Surrey, UK). pXC_50_ values were obtained using the ActivityBase XE nonlinear regression function in the full curve analysis bundle.

### Biochemical Assay of GuaB2 Inhibition

The *Mtb* ortholog of the enzyme was recombinantly expressed, purified and assayed as previously described^18^. Inhibition was determined at 0.1 mM, using 12 μg GuaB2 with the addition of 0.5% CHAPS. Each assay was normalised for DMSO concentration. Data was recorded in duplicate and analysed using Graphpad Prism 6.0.

### *In silico* molecular docking of Compounds JAGC_1–7

The Internal Coordinate Mechanism (ICM) method developed by Molsoft L.L.C^25^ was used to generate binding modes of the 9 small molecules in the binding pocket of selected GuaB2 crystal structures from the PDB and to estimate the strength of the protein-ligand interactions based on the ICM scoring function. The scoring function is defined as the sum of energy changes when the ligand binds to the protein, given as:


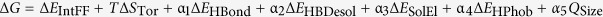


where Δ*E*_IntFF_, is the change in van der Waals interactions of ligand and receptor and the internal force-field energy of the ligand, *T*Δ*S*_Tor,_ is the change in free energy due to conformational entropy and weighted (α_1_ − α_5_), Δ*E*_HBond_ is the hydrogen bond term,

Δ*E*_HBDesol_ accounts for the disruption of hydrogen bonds with solvent, Δ*E*_SolEl_ is the solvation electrostatic energy change upon binding, Δ*E*_HPhob_ is the hydrophobic free energy gain and *Q*_size_ is the ligand size correction term. The ICM scores were standardized by calculating the ligand efficiency indices (LEI) as a ratio of the ICM score to the number of heavy atoms in the ligand for each docked molecule^26^.

### *In silico* protein structure preparation

A number of structures of GuaB2 have been deposited into the PDBe and four of these have GuaB2 co-crystallised with substrate, NAD^+^ and product, XMP (PDB code: 4zqm), and with IMP and antitubercular compounds (PDB codes: 4zqp, 4zqo and 4zqn). The structures reveal that NAD^+^ and XMP occupy different binding sites^19^ and the antitubercular compounds bind to the NAD^+^ pocket whilst IMP occupies the XMP site. The NAD^+^ site is an exposed open pocket defined by the residues N289, L291, V292, G260, T284, A285 and D283. This pocket extends into a tunnel-like pocket that forms part of the IMP binding site made up of residues like T343, G334, E458, N313, D374. We extracted the four crystal structures from the PBDe to use in our docking calculations. To validate our calculations, firstly, the coordinates of the ligands occupying the NAD^+^ pocket (akp3, a4qo, aq67 and NAD^+^) were removed from their respective structure files (Table 2) and were saved into separate files. Using ICM-docking receptor preparation tools the four protein structures were separately prepared by deleting all water molecules, optimize hydrogen, adding missing heavy atoms and hydrogen, and adjusting amide groups and were saved as ICM receptor molecules. The “setup receptor” tool was used to generate receptor maps using a grid size of 0.5 Å, and over binding sites consisting of 19 to 28 residues in protein structures (Table 2). Secondly, after extracting XMP from the 4ZQM structure, grid maps were generated over its binding site defined by 31 residues (Table 3). Overlaying their Cα atoms, calculating the root mean square deviations and analysing the residue side chain orientations in the binding pockets evaluated the differences between the 4 GuaB2 structures.

### Validation of docking calculations

The ligand structures were similarly prepared and converted to ICM molecules with the corrected bond order and stereochemistry. Cross-docking was used to assist with identifying the GuaB2 conformation and parameters to use during the production stage and to validate the docking calculations. Cross-docking involved docking each prepared ligands structures into the NAD^+^ binding pocket in all four GuaB2 conformations, including re-docking ligands into their respective protein structures and optimising the parameters. The generated binding conformations were compared to the crystal structure conformations. It was observed that the GuaB2 structure, PDB code: 4ZQP, was able to regenerate the crystal conformations of all ligands with root mean square deviations (RMSD) ranging from 0.94 to 2.00, indicating its versatility. Hence docking calculations using 4ZQP were repeated to optimise the docking parameters and the docking thoroughness/effort was set to 5. Similarly, the ligand XMP was re-docked into its binding pocket in structure 4ZQM and the docking parameters were optimised and thoroughness/effort was set to 2.

### *In silico* ligand preparation and docking

Three dimension coordinates of 9 compounds, including diphenyl urea, DPU-2, known to inhibit GuaB2, were generated using a Pipeline Pilot protocol and saved as mol2 files. The molecules were imported into ICMdock, amide bonds were fixed, hydrogen atoms were built and the structures were converted and saved as ICM compatible molecules. The compounds were first docked into the NAD^+^ binding pocket of 4ZPQ using thoroughness/effort value of 5 and default ICM parameters. To determine occupancy of the ligands in the large binding pocket, the compounds were re-docked into the XMP binding pocket in 4QZM. The best ICM scoring conformation for each compound was extracted and their ligand efficiency indices (LEI)^26^ were calculated dividing each ICM score by the number of heavy atoms in the molecules.

## Additional Information

**How to cite this article**: Cox, J. A. G. *et al*. Novel inhibitors of *Mycobacterium tuberculosis* GuaB2 identified by a target based high-throughput phenotypic screen. *Sci. Rep.* **6**, 38986; doi: 10.1038/srep38986 (2016).

**Publisher's note:** Springer Nature remains neutral with regard to jurisdictional claims in published maps and institutional affiliations.

## Supplementary Material

Supplementary Information

## Figures and Tables

**Table 1:** Z’ and pXC_50_ values for dose response validation of GuaB2 targeting compounds.

Z’ Value						
Target	Plate 1	Plate 2	Plate 3	Plate 4		
GuaB2	0.74	0.76	0.74	0.74		
GuaB2 EV	0.41	0.76	0.73	0.67		
**Compound ID**	**pXC**_**50**_ **for Empty Vector**	**pXC**_**50**_ **Mean for GuaB2**	**Shift pIC**_**50**_	**% Biochemical Inhibition of** ***Mtb*** **GuaB2**	**SD of % Biochemical Inhibition of** ***Mtb*** **GuaB2**	**MIC (μM) H37Rv**
JAGC_1	5.05	≤4	1.05	23.09	0.51	>80
JAGC_2	5.08	4.24	0.84	26.53	11.77	>80
JAGC_3	4.99	≤4	0.99	21.75	10.28	>80
JAGC_4	4.64	≤4	0.64	20.74	6.86	>80
JAGC_5	5.36	4.42	0.94	20.62	6.45	>100
JAGC_6	5.42	4.35	1.07	15.44	1.61	>80
JAGC_7	5.38	5.01	0.37	12.25	7.45	50
DPU-2				44.6	4.21	
INH				−8.88	2.64	

**Table 2:** ICM score for compounds JAGC_1-7 in the NAD^+^ and IMP binding sites of GuaB2.

JAGC code	MWt (g/mol)	NAD^+^ site ICM Score	NAD^+^ site LEI	IMP site ICM Score	IMP site LEI
JAGC_7	290.38	−19.72	0.99	−12.81	0.64
JAGC_2	372.85	−22.76	0.88	−1.41	0.05
JAGC_6	247.72	−13.53	0.8	0.22	−0.01
DPU_2	309.72	−16.36	0.78	−7.21	0.34
DPU_3	378.02	−12.82	0.64	−5.46	0.27
JAGC_1	362.43	−16.96	0.63	8.73	−0.32
JAGC_3	353.44	−13.52	0.56	−4.91	0.2
JAGC_4	327.83	−11.83	0.56	−3.57	0.17
JAGC_5	291.82	−9.77	0.49	3.96	−0.2
DPU_1	359.69	−9.01	0.38	5.51	−0.23
KP3	608.6	−19.1	0.43	Nd	nd
XMP	362.03	nd	Nd	−82.17	3.42

*nd = not determined, MWt = Molecular weight, LEI = Ligand Efficiency Index (ICM score/Number of heavy atoms), KP3 is the co-crystallised ligand in structure 4ZQP, XMP the product that occupies the IMP site in structure 4ZQM.

**Table 3:** Ligands and Grid properties for GuaB2 structures.

PDBe Structure Code	Ligand Code	Number of Binding site residues	Grid dimensions (X,Y,Z) (Å)	Grid size (Å)
4ZQP	KP3	28	16.31,18.39,15.64	0.5
4ZQO	Q67	19	23.43,21.81,23.89	0.5
4ZQM	NAD^+^	31	12.62,13.56,12.07	0.5
4ZQM	XMP	31	17.43,16.50,20.70	0.5
4ZQN	4ZQO	24	21.83,23.07,22.46	0.5
