# Supplementary material for: Novel inhibitors of Mycobacterium tuberculosis GuaB2 identified by a target based high-throughput phenotypic screen
Source: Sci Rep. 2016 Dec 16;6:38986. doi: 10.1038/srep38986 (PMC5159837; doi:10.1038/srep38986)
Supplement: Supplementary Information [file srep38986-s1.pdf]

Novel inhibitors of *Mycobacterium tuberculosis* GuaB2 identified by a target based high-throughput phenotypic screen

Jonathan A. G. Cox[1], Grace Mugumbate[2*], Laura Vela-Glez Del Peral[3], Monika Jankute[4], Katherine A. Abrahams[4], Peter Jervis[4], Stefan Jackenkroll[4], Arancha Perez[5], Carlos Alemparte[5], Jorge Esquivias[5], Joël Lelièvre[5], Fernando Ramon[3], David Barros[5], Lluis Ballell[5], Gurdyal S. Besra[4*]

[1] Life and Health Sciences, Aston University, Aston Triangle, Birmingham B4 7ET, UK

[2] European Molecular Biology Laboratory, European Bioinformatics Institute (EMBL-EBI), Wellcome Trust Genome Campus, Hinxton, Cambridge, United Kingdom, CB10 1SD

[3] Molecular Discovery Research, GlaxoSmithKline, Santiago Grisolia 4, 28760 Tres Cantos, Madrid, Spain

[4] School of Biosciences, University of Birmingham, Edgbaston, Birmingham B15 2TT, UK

[5] Diseases of the Developing World, GlaxoSmithKline, Severo Ochoa 2, 28760 Tres Cantos, Madrid, Spain

[*]Corresponding Authors: g.besra@bham.ac.uk; grace@ebi.ac.uk

Supplementary Table 1

	JAGC_1	JAGC_2	JAGC_3
**Chemical Nomenclature:**	N-(5-(3-(4-methylpiperazin-1-yl)quinoxalin-6-yl)pyridin-2-yl)acetamide	3-acetyl-N-(5-chloro-1,2,3,4-tetrahydroisoquinolin-7-yl)-4-ethoxybenzamide	methyl 5,5-dimethyl-2-(piperazine-1-carboxamido)-4,7-dihydro-5H-thieno[2,3-c]pyran-3-carboxylate
**Chemical Formula:**	C20H22N6O	C20H21ClN2O3	C16H23N3O4S
**Exact Mass:**	362.19	372.12	353.14
**Molecular Weight:**	362.44	372.85	353.44
**m/z:**	362.19 (100.0%), 363.19 (21.6%), 364.19 (2.2%), 363.18 (2.2%)	372.12 (100.0%), 374.12 (32.0%), 373.13 (21.6%), 375.12 (6.9%), 374.13 (2.2%)	353.14 (100.0%), 354.14 (17.3%), 355.14 (4.5%), 355.15 (1.4%), 354.14 (1.1%)
**Elemental Analysis:**	C, 66.28; H, 6.12; N, 23.19; O, 4.41	C, 64.43; H, 5.68; Cl, 9.51; N, 7.51; O, 12.87	C, 54.37; H, 6.56; N, 11.89; O, 18.11; S, 9.07
**Boiling Point:**	1071.05 [K]	1038.32 [K]	953.97 [K]
**Melting Point:**	902.19 [K]	822.08 [K]	845.84 [K]
**Gibbs Energy:**	870.09 [kJ/mol]	133.33 [kJ/mol]	19.7 [kJ/mol]
**Log P:**	1.92	2.46	-0.88
**MR:**	105.86 [cm3/mol]	104.41 [cm3/mol]	92.97 [cm3/mol]
**CLogP:**	2.27815	3.33859	1.56835

	JAGC_4	JAGC_5	JAGC_6
**Chemical Nomenclature:**	N-(7-chloro-4-methoxybenzo[d]thiazol-2-yl)-N-(2-(dimethylamino)ethyl)acetamide	(S)-2-chloro-4-(isopentyl(pyrrolidin-3-yl)amino)benzonitrile	3-chloro-1-(piperazin-1-yl)isoquinoline
**Chemical Formula:**	C14H18ClN3O2S	C16H22ClN3	C13H14ClN3
**Exact Mass:**	327.08	291.15	247.09
**Molecular Weight:**	327.83	291.82	247.73
**m/z:**	327.08 (100.0%), 329.08 (32.0%), 328.08 (15.1%), 330.08 (4.8%), 329.08 (4.5%), 331.07 (1.4%), 328.08 (1.1%), 329.09 (1.1%)	291.15 (100.0%), 293.15 (32.0%), 292.15 (17.3%), 294.15 (5.5%), 293.16 (1.4%), 292.15 (1.1%)	247.09 (100.0%), 249.08 (32.0%), 248.09 (14.1%), 250.09 (4.5%), 248.08 (1.1%)
**Elemental Analysis:**	C, 51.29; H, 5.53; Cl, 10.81; N, 12.82; O, 9.76; S, 9.78	C, 65.85; H, 7.60; Cl, 12.15; N, 14.40	C, 63.03; H, 5.70; Cl, 14.31; N, 16.96
**Boiling Point:**	817.22 [K]	817.66 [K]	724.73 [K]
**Melting Point:**	668.49 [K]	549.35 [K]	574.03 [K]
**Gibbs Energy:**	331.93 [kJ/mol]	530.84 [kJ/mol]	566.25 [kJ/mol]
**Log P:**	2.56	3.95	2.97
**MR:**	85.84 [cm3/mol]	85.58 [cm3/mol]	72.57 [cm3/mol]
**CLogP:**	2.60237	4.48298	2.60798

	JAGC_7
**Chemical Nomenclature:**	1-(benzo[d][1,2,3]thiadiazol-5-yl)-3-(4-methylcyclohexyl)urea
**Chemical Formula:**	C14H18N4OS
**Exact Mass:**	290.12
**Molecular Weight:**	290.39
**m/z:**	290.12 (100.0%), 291.12 (15.1%), 292.12 (4.5%), 291.12 (1.5%), 292.13 (1.1%)
**Elemental Analysis:**	C, 57.91; H, 6.25; N, 19.29; O, 5.51; S, 11.04
**Boiling Point:**	912.32 [K]
**Melting Point:**	769.98 [K]
**Gibbs Energy:**	499.58 [kJ/mol]
**Log P:**	3.89
**MR:**	82.04 [cm3/mol]
**CLogP:**	3.737
